# Social Cognitive Mediators of Sociodemographic Differences in Colorectal Cancer Screening Uptake

**DOI:** 10.1155/2015/165074

**Published:** 2015-10-04

**Authors:** Siu Hing Lo, Jo Waller, Charlotte Vrinten, Lindsay Kobayashi, Christian von Wagner

**Affiliations:** Department of Epidemiology and Public Health, Health Behaviour Research Centre, University College London, Gower Street, London WC1E 6BT, UK

## Abstract

*Background*. This study examined if and how sociodemographic differences in colorectal cancer (CRC) screening uptake can be explained by social cognitive factors. *Methods*. Face-to-face interviews were conducted with individuals aged 60–70 years (*n* = 1309) living in England as part of a population-based omnibus survey. *Results*. There were differences in screening uptake by SES, marital status, ethnicity, and age but not by gender. Perceived barriers (stand. *b* = −0.40, *p* < 0.001), social norms (stand. *b* = 0.33, *p* < 0.001), and screening knowledge (stand. *b* = 0.17, *p* < 0.001) had independent associations with uptake. SES differences in uptake were mediated through knowledge, social norms, and perceived barriers. Ethnic differences were mediated through knowledge. Differences in uptake by marital status were primarily mediated through social norms and to a lesser extent through knowledge. Age differences were largely unmediated, except for a small mediated effect via social norms. *Conclusions*. Sociodemographic differences in CRC screening uptake were largely mediated through social cognitive factors. *Impact*. Our findings suggest that multifaceted interventions might be needed to reduce socioeconomic inequalities. Ethnic differences might be reduced through improved screening knowledge. Normative interventions could emphasise screening as an activity endorsed by important others outside the immediate family to appeal to a wider audience.

## 1. Introduction

Colorectal cancer (CRC) screening using a guaiac-based faecal occult blood test (gFOBt) lowers CRC mortality by up to 25% among those who participate [1, 2]. The National Health Service (NHS) Bowel Cancer Screening Programme (BCSP) sends all age-eligible (60–69, recently extended to 74) men and women living in England a free home-based gFOB test every two years, usually starting from their 60th birthday. Patient data from General Practitioner (GP) lists are used to approach eligible adults, so over 95% of the national population in the eligible age range is invited [3]. The test involves taking three stool samples and returning the kit to the laboratory in a freepost envelope. Despite the lack of financial barriers to screening, low and socially unequal uptake has been a persistent public health concern since the screening programme was introduced in 2006 [3, 4].

Socioeconomic status (SES) has consistently been associated with CRC screening uptake across healthcare systems [5, 6]. For instance, uptake of first-time screening invitations in England ranged from 35% in the most deprived quintile to 61% in the most affluent quintile of areas in the country [3]. Ethnic differences in CRC screening have also been frequently observed and appear to be independent of or only partially explained by other sociodemographic factors [7–9]. Although first-time gFOB test uptake is around 8% higher among women than men, gender differences in uptake are less persistent over time than SES effects [4, 10]. Being married has been related to higher uptake of other CRC screening modalities [11, 12], although few studies have examined the role of marital status in the context of gFOBt screening specifically [13].

In parallel with studies of the sociodemographic patterning of CRC screening behaviour, psychological models, such as the Health Belief Model (HBM) [14] and Theory of Planned Behaviour (TPB) [15], have been used to investigate social cognitive factors such as attitudes, knowledge, social norms, and perceived barriers to screening [5, 16, 17]. Social cognitive factors are typically measured using questions about relevant beliefs and are generally viewed as more proximal and modifiable determinants of behaviour than sociodemographic factors [18]. Social cognitive factors would therefore be expected to mediate the association between sociodemographic factors and health behaviours [15].

General attitudes towards CRC screening in the population have been found to be very positive [19]. Nevertheless, there is relatively low awareness of CRC as a common cancer [20]. Misconceptions about the purpose of CRC screening, such as the belief that screening is only needed if one has symptoms, are also commonly reported among non-responders to screening [21–23]. The disgust, embarrassment and practicalities of stool sampling are well-documented barriers to gFOB screening [21, 23–25], and difficulty overcoming such perceived barriers is another common reason given for not taking part in CRC screening [13]. Furthermore, social norms have also been consistently related to CRC screening [16]. Due to the home-based nature of the gFOB test and the lack of direct contact with health professionals, it is plausible that any normative influence from nonmedical sources might be particularly relevant to CRC screening in the organised screening programme in England.

Although a framework has been developed to summarise potential social cognitive mediators of socioeconomic inequalities in screening uptake [26], few studies have empirically examined these pathways using mediation modelling. One study examining CRC and prostate screening in men showed that sociodemographic differences in screening uptake were largely attributable to the TPB-based social cognitive constructs (attitude, perceived norms, and perceived behavioural control) [27], but none has explored the specific pathways through which each sociodemographic variable affects uptake. Understanding these social cognitive pathways may help the development of effective and targeted interventions to reduce sociodemographic inequalities in cancer screening.

In this study, we aimed to explore social cognitive mediators of sociodemographic differences in gFOBt screening uptake in England. The objectives were to explore the associations between sociodemographic factors and gFOBt uptake in a cross-sectional dataset and to test mediation models exploring potential social cognitive mechanisms underlying sociodemographic differences in uptake, with a view to developing hypotheses to test in future prospective studies.

## 2. Methods

The data were collected as part of a TNS Research International population-based omnibus survey conducted in Great Britain between January and March 2014. Each week, up to 4000 people (aged 16+) are interviewed for the omnibus survey. The TNS omnibus survey defines sampling points using 2001 Census small-area statistics and the Postcode Address File (stratified by social grade and Government Office Region) for random location sampling selection. Response rates are not recorded. However, at each location, quotas are set for age, sex, children in the home, and working status to ensure a sample that reflects the demographic characteristics of the national population. Survey respondents were asked to take part in face-to-face interviews using computer-assisted personal interviewing (CAPI) on a voluntary basis. Only respondents aged 58–70 were included in the section of the omnibus survey about cancer screening.

### 2.1. Participants

Responses were collected from 1568 men and women living in England aged 58–70 years with no CRC history. One hundred eighty-seven respondents were excluded from the present analysis because they were aged between 58 and 59 and therefore not yet eligible for CRC screening at the time of the interview, leaving 1381 eligible respondents. This ensured that the included respondents should have been invited for CRC screening through the organised national programme, regardless of whether they remembered having been invited or not. Seventy-two respondents (5%) who had missing values (i.e., “refused” or “don't know”) for the outcome variable “screening uptake” were also excluded. The final sample included 1309 respondents (95% of those eligible).

### 2.2. Measures

*Screening Uptake.* Respondents were asked if they had ever been invited to do a stool test for the NHS BCSP. If they had been invited, they were asked further questions about how many times they had been invited and how many times they had taken part. Self-report of not having been invited is likely due to reasons other than truly not having been sent a screening invitation (e.g., not remembering the invitation) because included respondents were eligible for screening through the national CRC screening programme in England. A dummy variable for screening uptake therefore coded respondents as nonresponders (not invited* OR* no test kits completed) or ever responders (≥1 test kit completed). 

*Social Cognitive Factors*. Social cognitive measures were informed by previous literature on CRC screening uptake and social cognitive models of behaviour. Belief in the usefulness of asymptomatic screening [21–23] was measured in lieu of general attitude towards screening due to known ceiling effects in screening attitude [19]. An injunctive norm measure (i.e., what other people think one should do) and a descriptive norm measure (i.e., what other people do themselves) were included to be consistent with the social norms literature [28]. Finally, the most salient emotional [23–25] and time/delay [13, 21, 29] barriers known to be associated with poorer CRC screening uptake were used as measures of the perceived barriers factor. Respondents rated the extent of their (dis)agreement with a series of belief statements on a five-point scale (strongly agree/lightly agree/neither agree nor disagree/slightly disagree/strongly disagree).

*Screening knowledge* was measured with the statement “People only need to take part in bowel cancer screening if they have symptoms” and was reverse-coded.

*Social norms* were measured using one injunctive norm statement, “People who are important to me think that I should take part in bowel cancer screening,” and one descriptive norm statement, “People who are important to me take part in bowel cancer screening.”

*Perceived barriers* were measured with two statements measuring the respondent's ability to overcome emotional barriers: “It is difficult to overcome the disgust related to the stool test” and “it is difficult to overcome the embarrassment related to the stool test.” A third statement was used to measure ability to overcome practical time barriers “It is difficult to get round to doing the stool test.” 

*Sociodemographic Variables*. Age, gender, marital status (married/divorced, separated, or widowed/single), ethnicity (white/nonwhite), and SES (A/B/C1/C2/D/E) were measured. The ordinal measure of SES was based on the National Readership Survey social grade classification system which ranks people according to occupation (or previous occupation if retired): A (higher managerial, administrative, or professional), B (intermediate managerial, administrative, or professional), C1 (supervisory, clerical or junior managerial, administrative, or professional), C2 (skilled manual), D (semiskilled or unskilled manual), or E (state pensioners, casual/lowest grade workers, or unemployed with state benefits only). The occupational status of the household's chief wage earner was used to assess SES if the respondent was not working.

### 2.3. Data Analysis

Screening uptake was first analysed by sociodemographic groups (Table 2) and by social cognitive beliefs (Table 3). The multivariable associations between sociodemographic variables and screening uptake were examined using logistic regression analysis (Table 2). Sociodemographic variables that were significantly associated with uptake (*p* < 0.05) were included in a multivariable analysis. Bivariate associations between social cognitive beliefs and uptake were also examined with logistic regression (Table 3). All logistic regression analyses were conducted using Stata SE13 [30].

Mediation of sociodemographic effects on screening uptake via social cognitive factors was then tested using Structural Equation Modelling (SEM) with MPlus 7.11 [31]. Hu and Bentler's guidelines for goodness-of-fit were used, with statistics around 0.95 and above for the Comparative Fit Index (CFI) and Tucker-Lewis Index (TLI), and around 0.08 and below for the Root Mean Square Error of Approximation (RMSEA) and Standard Root Mean Residual (SRMR) deemed as indicators of good fit [32].

Before testing for mediation, two models were first tested to assess goodness-of-fit of the measurement model and the path model with social cognitive factors as predictors of uptake. The measurement model (Model I) of the social cognitive factors with measures grouped as described above was tested using Confirmatory Factor Analysis (CFA) (Table 4). As most indicators were nonnormally distributed MLM, a robust maximum likelihood estimator, was used to obtain estimates. The hypothesised measurement model showed adequate fit. The measurement model could therefore be extended into a SEM model by including uptake as the outcome variable. WLSMV, a robust weighted least squares estimator, which is the default estimator for binary outcome models in MPlus, was used for all SEM models. The first SEM model (Model II) tested for direct effects of social cognitive factors on uptake. Social cognitive factors were allowed to correlate freely with each other because they were strongly correlated and no hypotheses regarding the relationships between social cognitive factors needed to be tested. Similar to Model I, Model II had adequate goodness-of-fit statistics, indicating that all social cognitive factors had direct, independent effects on uptake as expected. Therefore, subsequent SEM models included direct paths from all social cognitive factors to uptake.

**Figure 1:**
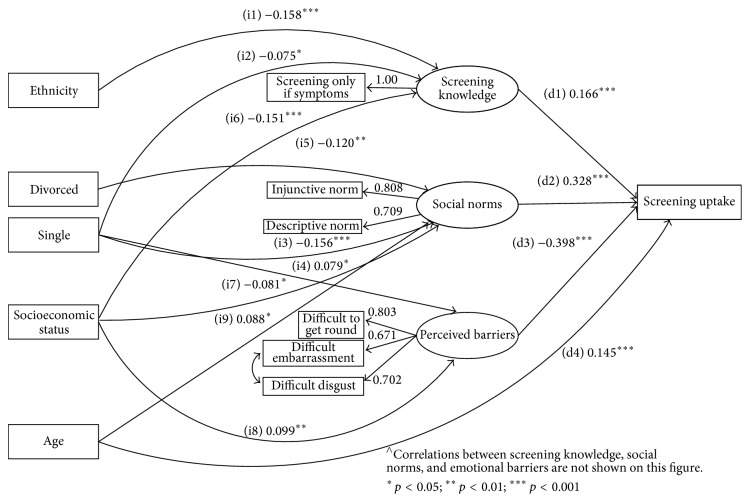
Full mediation of sociodemographic differences in screening uptake via social cognitive factors (Model IV, Table 3)  ^∧^.

Model III aimed to test mediation of sociodemographic differences in uptake via social cognitive factors. The model included (1) direct paths from social cognitive factors to uptake; (2) direct paths from sociodemographic variables to uptake; and (3) indirect paths from sociodemographics via social cognitive factors to uptake. For the sake of parsimony, nonsignificant paths in Model III were removed using stepwise backward elimination to obtain the final model (Model IV, Figure 1). As in Model II, Models III and IV allowed all social cognitive factors to freely correlate with each other. Sociodemographic variables were not correlated, as they were largely independent predictors of uptake (Table 2).

To compare Model III with Model IV, the models were first run without bootstrapping to obtain chi-square statistics for a chi-square of difference test for models using the WLSMV estimator (DIFFTEST option in MPlus). The same models were estimated again with bootstrapping to obtain robust standard errors and confidence intervals of the point estimates. Bootstrapping is recommended for mediation analysis because the method tends to have the best statistical power and Type I error control [33]. Bootstrapping with 10,000 resamplings of the dataset was used to obtain bias-corrected confidence intervals. *p* values from the bootstrapped model estimates were reported. Standardized path coefficients are reported for the final model (Model IV) to aid interpretation of the probit regression coefficients provided by the WLSMV estimator. Standardized indirect effects of sociodemographic variables on uptake are also reported.

## 3. Results

Of the total included samples, 50.7% were men (Table 1). The age range of the included sample was 60–70 with a mean age of 65 (SD = 3.2). The majority of respondents (65.0%) were married, 26.2% were divorced, separated, or widowed, and 8.8% were single. The socioeconomic distribution of the sample was as follows: 5.0% in A (the highest grade), 20.4% in B, 22.1% in C1, 18.3% in C2, 11.6% in D, and 22.5% in E (the lowest grade). Only 4.1% (*n* = 53) of respondents were nonwhite which reflects the low prevalence of ethnic minorities among older age groups in the national population of England [34].

Overall, 69.4% of respondents reported having taken part in screening at least once (Table 2). Of the respondents who had never participated (*n* = 401), 50.1% (*n* = 201) indicated they had never been invited.

### 3.1. Sociodemographics and Screening Uptake

Gender was not associated with screening uptake in the sample (Table 2). SES was associated with uptake in a graded fashion, from 59.3% in the lowest grade E to 74.2% in the highest grade A (*p* < 0.001). Nonwhite respondents were also less likely ever to have responded to screening invitations than white respondents (41.5% versus 70.5%, *p* < 0.001). Single people had lower uptake than those who were married (55.7% versus 71.7%, *p* < 0.001). The difference in uptake rates between being divorced, separated, or widowed and being married was not statistically significant (68.2% versus 71.7%, ns.). Older age was also associated with higher uptake (60–64: 62.6% versus 65–70: 74.3%, *p* < 0.001). A multivariable logistic regression analysis showed that all significant sociodemographic predictors were associated with uptake independently of one another (Table 2).

### 3.2. Social Cognitive Factors and Screening Uptake

The prevalence of social cognitive beliefs and their association with uptake are described in Table 3. Bivariate analysis showed that all social cognitive measures were significantly associated with uptake (all *p* < 0.001). A CFA analysis confirmed that the measurement model had a good fit (Model I, Table 4). This suggests that the belief statements were related to their respective social cognitive factor (screening knowledge, social norms, or perceived barriers) as described in Section 2. All latent factors were correlated (screening knowledge with social norms: *r* = −0.43; screening knowledge with perceived barriers: *r* = 0.49; and social norms with perceived barriers: *r* = −0.41, all *p* < 0.001). A SEM model with direct paths from each social cognitive factor to uptake (and correlated latent factors) also had an adequate fit (Model II).

### 3.3. Social Cognitive Mediation of Sociodemographic Differences in Uptake

A SEM model with both direct and indirect paths from SES, ethnicity, marital status, and age showed adequate fit statistics. However, the direct paths from SES, ethnicity, and marital status to screening uptake were not statistically significant (Model III, Table 4). Age was the only sociodemographic variable with a significant direct path to screening uptake. A final SEM model preserved significant indirect paths from SES, ethnicity, marital status, and age to uptake and direct paths from social cognitive factors and age to uptake (Model IV, Table 4; Figure 1). A chi-square difference test for the WLSMV estimator showed that the more parsimonious Model IV did not have a significantly worse fit than Model III (Δ*χ* ^2^ = 9.033, Δdf = 9, *p* = 0.43).

In the final model (Model IV, Figure 1), screening knowledge (d1), social norms (d2), perceived barriers (d3), and age (d4) had direct effects on uptake (all *p* < 0.001). Ethnicity had a significant indirect path to uptake via screening knowledge ([i1] white: ref. cat.; nonwhite: stand. ind. effect = −0.027, 95% CI: −0.045–−0.010, *p* < 0.01). Marital status had a stronger indirect path to uptake via social norms (married: reference category; single [i3]: stand. ind. effect = −0.047, 95% CI: −0.072–−0.021, *p* < 0.001; divorced [i5]: stand. ind. effect = −0.037, 95% CI: −0.062–−0.011, *p* < 0.01) and a weaker indirect path via screening knowledge (single [i2]: stand. ind. effect = −0.012, 95% CI: −0.024–−0.001, *p* < 0.05) and perceived barriers (single [i4]: stand. ind. effect = 0.032, 95% CI: −0.063–−0.001, *p* < 0.05). SES had significant indirect paths to uptake via screening knowledge ([i6] stand. ind. effect = −0.026, 95% CI: −0.041–−0.011, *p* < 0.001), social norms ([i7] stand. ind. effect = −0.025, 95% CI: −0.049–−0.001, *p* < 0.05), and perceived barriers ([i8] stand. ind. effect = −0.040, 95% CI: −0.070–−0.009, *p* < 0.05). Age had a significant indirect path to uptake via social norms ([i9] stand. ind. effect = 0.027, 95% CI: 0.005–0.049, *p* < 0.05).

## 4. Discussion

The present study explored social cognitive mechanisms underlying sociodemographic differences in uptake of CRC screening using gFOBt in England. Of the three social cognitive factors, perceived barriers and social norms were most strongly associated with uptake, while screening knowledge showed a weaker association. The relatively strong associations of perceived barriers and social norms with uptake suggest that changes in beliefs related to these social cognitive factors might result in the largest impact on overall screening uptake.

### 4.1. Social Cognitive Mediators of Sociodemographic Differences

Mediation of sociodemographic differences in uptake via screening knowledge, social norms, and perceived barriers was tested. SES differences in uptake were mediated through all three social cognitive factors, while ethnic differences in uptake were mediated via screening knowledge alone. Differences in uptake by marital status were primarily mediated through social norms and to a lesser extent through screening knowledge and perceived barriers. Age had a direct, positive effect on uptake and a smaller indirect effect via social norms. Overall, these findings indicate that, with the exception of age, sociodemographic differences in uptake may be largely mediated via social cognitive factors derived from psychological models such as the TPB [15] and the HBM [14].

#### 4.1.1. Mediation of Socioeconomic Differences in Screening Uptake

The current findings also suggest that socioeconomic inequalities in screening uptake are multidimensional and are unlikely to be entirely resolved through changes in one or a few key beliefs. This study suggests that social cognitive factors derived from common psychological models are mediators of socioeconomic difference in CRC screening uptake. This extends on previous research, which has demonstrated mediation of socioeconomic differences in screening uptake via cancer-specific social cognitive constructs, such as cancer worry [35] and fatalism [36]. If our findings are confirmed in longitudinal studies, interventions may need to target a range of beliefs, including those related to perceived barriers, screening knowledge, and social norms. However, a single, well-designed intervention might be able to target several relevant beliefs simultaneously, given that they tend to be correlated. Stepped interventions which offer generic educational material and advice to all and more tailored assistance for persistent nonresponders seem a promising intervention format for this purpose [37, 38].

#### 4.1.2. Mediation of Ethnic Differences in Screening Uptake

In line with previous research on cervical screening uptake among ethnic minority women [39], ethnic differences in CRC screening uptake were solely mediated via screening knowledge. Awareness campaigns targeted towards specific ethnic minority groups could therefore be useful. Although the English CRC screening programme already provides written translations of their information booklet, more could be done to engage people from ethnic groups who do not respond well to written information, even if provided in their native tongue [40].

#### 4.1.3. Mediation of Differences in Screening Uptake by Marital Status

Marital status appears to influence screening uptake primarily through social norms, possibly due to the availability of a clearly defined referent group for married people (i.e., their spouse) and by implication more salient social norms. Differences in uptake by marital status could be caused or inadvertently aggravated if normative messages focus on partners or children as a reason to take part in screening. Public communication should acknowledge that roughly one-third of the target group for cancer screening do not or no longer have a life partner or children. Other potential sources of normative messages, such as health care providers and community leaders, could appeal to a wider audience, including those without a partner or children. The overall findings for marital status suggest that a life partner can highlight the social relevance of screening as well as increasing relevant knowledge and reducing perceived barriers to screening, albeit it to a lesser extent. This is consistent with previous qualitative findings which emphasised the influence of talking about screening and being aware of one's partner's or friends' screening participation on uptake [21].

#### 4.1.4. Mediation of Age Differences in Screening Uptake

Although age differences in screening uptake were largely unmediated via social cognitive factors in the tested model, a small indirect effect via social norms might indicate that people are gradually exposed to more positive norms as they age (although it could also be a cohort effect). This might have a positive impact on screening uptake among those who have not responded to earlier screening invitations [21]. In the present study, the outcome measure was whether respondents had “ever” participated in screening, which is likely to be associated with age, simply by virtue of the fact that older people will have received more invitations and therefore had more opportunities to participate. Future research should examine if these observed age effects on uptake also extend to other screening uptake outcomes (e.g., regular uptake over time).

### 4.2. Study Limitations

The present study results should be interpreted in the light of widely discussed limitations of using cross-sectional data for mediation analyses [41, 42]. Although our findings are plausible and are consistent with psychological theory and a number of previous findings, it is essential that they are replicated using longitudinal methods in order to confirm the mediation effects. This survey provided the opportunity to explore associations in a large population-based sample, but our findings must be treated with caution and should be used to develop hypotheses for future studies.

Another study limitation was that screening knowledge was measured with a single item (“People only need to take part in bowel cancer screening if they have symptoms”). Although the measure taps into a common misconception about screening which has direct implications for screening participation, the results might not generalise to other knowledge measures. Findings regarding ethnic differences in uptake should also be replicated in studies with a larger sample of nonwhite respondents, as the ethnic minority sample was small and is unlikely to have been representative of all ethnic minority groups in England.

A final limitation is our inability to report a response rate due to the methods used by the survey company that carried out the fieldwork. Although this means that we cannot rule out participation bias, it is unlikely that attitudes to screening would have been associated with participation, as the survey was part of an omnibus, including questions on a wide range of subjects. The sampling method ensured that the demographic profile of the sample was broadly similar to the national population.

## 5. Conclusion

In conclusion, the present study has identified possible social cognitive pathways through which sociodemographic factors could affect colorectal cancer screening uptake. A range of social cognitive factors seem to be associated with socioeconomic inequalities, whereas only lack of screening knowledge was associated with ethnic inequalities. Social norms were the main mediator of uptake differences by marital status. The study findings could inform the development of hypotheses to be tested in future longitudinal studies, with a view to developing interventions aimed at reducing sociodemographic differences in CRC screening uptake.

## Figures and Tables

**Table 1:** Sociodemographic characteristics of the included sample.

	Sample characteristics
	% (*n*)
Total	100% (1309)

Gender	
Men	50.7% (664)
Women	49.3% (645)
Marital status	
Married	65.0% (851)
Divorced, separated, or widowed	26.2% (343)
Single	8.8% (115)
Ethnicity	
White	95.9% (1256)
Nonwhite	4.1% (53)
Socioeconomic status (A–E)	
A	5.0% (66)
B	20.4% (267)
C1	22.1% (289)
C2	18.3% (240)
D	11.6% (152)
E	22.5% (295)
Age (60–70)	
60–64	42.1% (551)
65–70	57.91% (758)

**Table 2:** Sociodemographic characteristics and screening uptake: descriptive statistics and logistic regression results.

	Screening uptake By sociodemographic group % (*n*)	Adjusted Odds Ratios (OR) With screening uptake as outcome	
		Multivariable logistic regression results	
		OR	95% CI
Total	69.4% (1309)		

Gender			
Men	69.1% (664)	(ref.)	
Women	69.6% (645)^a^	1.03	0.81–1.32
Marital status			
Married	71.7% (851)	(ref.)	
Divorced, separated, or widowed	68.2% (343)^a^	0.97	0.73–1.29
Single	55.7% (115)^a*∗∗*^	0.57^*∗∗*^	0.38–0.86
Ethnicity			
White	70.5% (1256)	(ref.)	
Non-white	41.5% (53)^a*∗∗∗*^	0.34^*∗∗∗*^	0.19–0.60
Socioeconomic status (A–E)		0.85^b*∗∗∗*^	0.78–0.92
A	74.2% (66)		
B	76.0% (267)		
C1	74.1% (289)		
C2	71.3% (240)		
D	63.2% (152)		
E	59.3% (295)^a*∗∗∗*^		
Age (60–70)		1.08^b*∗∗∗*^	1.04–1.12
60–64	62.6% (551)		
65–70	74.3% (758)		

^a^bivariate regression results, ^b^as a continuous variable in the logistic regression analysis.

^*∗∗*^ *p* < 0.01; ^*∗∗∗*^ *p* < 0.001.

**Table 3:** Social cognitive beliefs and screening uptake: descriptive statistics and logistic regression results.

	Social cognitive beliefs % (*n*)	Screening uptake	Screening uptake	
		By agreement with social cognitive beliefs	Bivariate logistic regression results Using social cognitive beliefs as continuous variables (1–5)	
		% (*n*)	OR	95% CI
People only need to take part if they have symptoms^a^			3.46^*∗∗∗*^	2.68–4.46
Neither agree nor disagree, slightly or strongly disagree	86.0% (1091)	76.8% (1091)		
Slightly or strongly agree	14.0% (178)	37.6% (178)		
Difficult to get round to doing the test			0.48^*∗∗∗*^	0.43–0.53
Neither agree nor disagree, slightly or strongly disagree	84.7% (1072)	76.9% (1072)		
Slightly or strongly agree	15.3% (193)	42.0% (193)		
Difficult to overcome the embarrassment			0.52^*∗∗∗*^	0.47–0.58
Neither agree nor disagree, slightly or strongly disagree	87.3% (1110)	75.0% (1110)		
Slightly or strongly agree	12.7% (161)	46.0% (161)		
Difficult to overcome the disgust			0.50^*∗∗∗*^	0.44–0.56
Neither agree nor disagree, slightly or strongly disagree	88.5% (1124)	75.8% (1124)		
Slightly or strongly agree	11.5% (146)	37.0% (146)		
People who are important to me think I should take part			2.06^*∗∗∗*^	1.84–2.31
Neither agree nor disagree, slightly or strongly disagree	30.3% (367)	49.3% (367)		
Slightly or strongly agree	69.7% (846)	81.7% (846)		
People who are important to me take part			1.77^*∗∗∗*^	1.60–1.96
Neither agree nor disagree, slightly or strongly disagree	32.5% (384)	51.3% (384)		
Slightly or strongly agree	67.5% (797)	82.1% (797)		

^a^reverse coded for the logistic regression analysis.

^*∗∗∗*^ *p* < 0.001.

**Table 4:** CFA and SEM models: goodness-of-fit statistics (*n* = 1121).

	Model I	Model II	Model III	Model IV Figure 1
	CFA model with social cognitive factors	SEM model with direct paths from social cognitive factors to uptake	Model II plus all direct and indirect paths from sociodemographics to uptake	Model III excluding all nonsignificant paths

Estimator	MLM	WLSMV	WLSMV	WLSMV

*χ* ^2^	11.725	23.797	29.627	35.219
df	6	9	25	34
CFI	0.996	0.983	0.996	0.999
TLI	0.991	0.961	0.991	0.998
RMSEA	0.029	0.038	0.013	0.006
SRMR	0.014	—		—
WRMR	0.558	0.308	0.364	0.559

## References

[1] Hewitson P., Glasziou P., Watson E., Towler B., Irwig L. (2008). Cochrane systematic review of colorectal cancer screening using the fecal occult blood test (Hemoccult): an update. *American Journal of Gastroenterology*.

[2] Scholefield J. H., Moss S., Sufi F., Mangham C. M., Hardcastle J. D. (2002). Effect of faecal occult blood screening on mortality from colorectal cancer: results from a randomised controlled trial. *Gut*.

[3] von Wagner C., Baio G., Raine R. (2011). Inequalities in participation in an organized national colorectal cancer screening programme: results from the first 2.6 million invitations in England. *International Journal of Epidemiology*.

[4] Lo S. H., Halloran S., Snowball J., Seaman H., Wardle J., von Wagner C. (2014). Colorectal cancer screening uptake over three biennial invitation rounds in the English bowel cancer screening programme. *Gut*.

[5] Gregory T. A., Wilson C., Duncan A., Turnbull D., Cole S. R., Young G. (2011). Demographic, social cognitive and social ecological predictors of intention and participation in screening for colorectal cancer. *BMC Public Health*.

[6] Neter E., Stein N., Barnett-Griness O., Rennert G., Hagoel L. (2014). From the bench to public health: population-level implementation intentions in colorectal cancer screening. *The American Journal of Preventive Medicine*.

[7] Szczepura A., Price C., Gumber A. (2008). Breast and bowel cancer screening uptake patterns over 15 years for UK south Asian ethnic minority populations, corrected for differences in socio-demographic characteristics. *BMC Public Health*.

[8] Liss D. T., Baker D. W. (2014). Understanding current racial/ethnic disparities in colorectal cancer screening in the United States: the contribution of socioeconomic status and access to care. *American Journal of Preventive Medicine*.

[9] Robb K. A., Power E., Atkin W., Wardle J. (2008). Ethnic differences in participation in flexible sigmoidoscopy screening in the UK. *Journal of Medical Screening*.

[10] Moss S. M., Campbell C., Melia J. (2012). Performance measures in three rounds of the English bowel cancer screening pilot. *Gut*.

[11] Stimpson J. P., Wilson F. A., Watanabe-Galloway S., Peek M. K. (2012). The effect of marriage on utilization of colorectal endoscopy exam in the United States. *Cancer Epidemiology*.

[12] van Jaarsveld C. H. M., Miles A., Edwards R., Wardle J. (2006). Marriage and cancer prevention: does marital status and inviting both spouses together influence colorectal cancer screening participation?. *Journal of Medical Screening*.

[13] Lo S. H., Waller J., Wardle J., von Wagner C. (2013). Comparing barriers to colorectal cancer screening with barriers to breast and cervical screening: a population-based survey of screening-age women in Great Britain. *Journal of Medical Screening*.

[14] Rosenstock I. M. (1966). Why people use health services. *The Milbank Memorial Fund Quarterly: Health and Society*.

[15] Fishbein M., Ajzen I. (2010). *Predicting and Changing Behavior: The Reasoned Action Approach*.

[16] Kiviniemi M. T., Bennett A., Zaiter M., Marshall J. R. (2011). Individual-level factors in colorectal cancer screening: a review of the literature on the relation of individual-level health behavior constructs and screening behavior. *Psycho-Oncology*.

[17] Power E., Miles A., von Wagner C., Robb K., Wardle J. (2009). Uptake of colorectal cancer screening: system, provider and individual factors and strategies to improve participation. *Future Oncology*.

[18] Armitage C. J., Conner M. (2000). Social cognition models and health behaviour: a structured review. *Psychology and Health*.

[19] Taskila T., Wilson S., Damery S. (2009). Factors affecting attitudes toward colorectal cancer screening in the primary care population. *British Journal of Cancer*.

[20] Juszczyk D., Simon A. E., Waller J., Ramirez A. J., Wardle J. (2011). Do the UK public realise that colorectal cancer is a common cancer?. *Colorectal Disease*.

[21] Palmer C. K., Thomas M. C., Von Wagner C., Raine R. (2014). Reasons for non-uptake and subsequent participation in the NHS Bowel cancer screening programme: a qualitative study. *British Journal of Cancer*.

[22] Clavarino A. M., Janda M., Hughes K. L. (2004). The view from two sides: a qualitative study of community and medical perspectives on screening for colorectal cancer using FOBT. *Preventive Medicine*.

[23] Chapple A., Ziebland S., Hewitson P., McPherson A. (2008). What affects the uptake of screening for bowel cancer using a faecal occult blood test (FOBt): a qualitative study. *Social Science and Medicine*.

[24] Consedine N. S., Ladwig I., Reddig M. K., Broadbent E. A. (2011). The many faeces of colorectal cancer screening embarrassment: preliminary psychometric development and links to screening outcome. *British Journal of Health Psychology*.

[25] O'Sullivan I., Orbell S. (2004). Self-sampling in screening to reduce mortality from colorectal cancer: a qualitative exploration of the decision to complete a faecal occult blood test (FOBT). *Journal of Medical Screening*.

[26] von Wagner C., Good A., Whitaker K. L., Wardle J. (2011). Psychosocial determinants of socioeconomic inequalities in cancer screening participation: a conceptual framework. *Epidemiologic Reviews*.

[27] Sieverding M., Matterne U., Ciccarello L. (2010). What role do social norms play in the context of men's cancer screening intention and behavior? Application of an extended theory of planned behavior. *Health Psychology*.

[28] Schultz P. W., Nolan J. M., Cialdini R. B., Goldstein N. J., Griskevicius V. (2007). The constructive, destructive, and reconstructive power of social norms. *Psychological Science*.

[29] Lo S. H., Halloran S., Snowball J., Seaman H., Wardle J., von Wagner C. (2014). Predictors of repeat participation in the NHS bowel cancer screening programme. *British Journal of Cancer*.

[30] StataCorp (2013). *Stata Statistical Software: Release 13*.

[31] Muthén L. K., Muthén B. O. (1998–2012). *Mplus User's Guide*.

[32] Hu L.-T., Bentler P. M. (1999). Cutoff criteria for fit indexes in covariance structure analysis: conventional criteria versus new alternatives. *Structural Equation Modeling*.

[33] Hayes A. F. (2009). Beyond Baron and Kenny: statistical mediation analysis in the new millennium. *Communication Monographs*.

[34] Office of National Statistics (2005). *Focus on Ethnicity and Identity*.

[35] Wardle J., McCaffery K., Nadel M., Atkin W. (2004). Socioeconomic differences in cancer screening participation: comparing cognitive and psychosocial explanations. *Social Science and Medicine*.

[36] Miles A., Rainbow S., von Wagner C. (2011). Cancer fatalism and poor self-rated health mediate the association between socioeconomic status and uptake of colorectal cancer screening in England. *Cancer Epidemiology Biomarkers & Prevention*.

[37] Baker D. W., Brown T., Buchanan D. R. (2014). Comparative effectiveness of a multifaceted intervention to improve adherence to annual colorectal cancer screening in community health centers: a randomized clinical trial. *JAMA Internal Medicine*.

[38] Green B. B., Wang C.-Y., Anderson M. L. (2013). Automated intervention with stepped increases in support to increase uptake of colorectal cancer screening: a randomized trial. *Annals of Internal Medicine*.

[39] Ackerson K., Gretebeck K. (2007). Factors influencing cancer screening practices of underserved women. *Journal of the American Academy of Nurse Practitioners*.

[40] Team UCSPEE (2003). *Ethnicity: UK Colorectal Cancer Screening Pilot*.

[41] Maxwell S. E., Cole D. A. (2007). Bias in cross-sectional analyses of longitudinal mediation. *Psychological Methods*.

[42] Maxwell S. E., Cole D. A., Mitchell M. A. (2011). Bias in cross-sectional analyses of longitudinal mediation: partial and complete mediation under an autoregressive model. *Multivariate Behavioral Research*.

